# Environmental tobacco smoke exposure among non-smoking waiters: measurement of expired carbon monoxide levels

**DOI:** 10.1590/S1516-31802000000400001

**Published:** 2000-07-07

Despite the fact that tobacco use continues to be a public health problem in developed countries, there has been growth in its impact on mortality profiles in developing countries. Some nations of Latin America are among these countries, highlighting Chile, Uruguay, Argentina and Brazil.^1^ However, it is not only the smokers who are responsible for this situation, but also the passive smokers, those who do not smoke but breathe and live with cigarette smoke in closed spaces.

The long-term effect of exposure to ETS - Environmental Tobaco Smoke - has been the target of innumerable studies over the last 20 years. Consistent epidemiological evidence has been accumulated for an association between such exposure and lung cancer and atherosclerotic cardiovascular disease, as well as respiratory signs and symptoms, among other effects.^2,3,4^

This evidence has been contested by the tobacco industry, both in relation to its scientific content,^5^ and the actions that society has been adopting for its control.^6^ Such contestation has created obstacles to better social approaches to the problem.

Nevertheless, the impact of passive smoking on the health of the general population has repercussions that require immediate action, because despite the lower relative risk of tobacco-related diseases for passive smokers in relation to smokers, it can be presumed that the population exposed to ETS is greater than that directly exposed to tobacco abuse. This implies a greater absolute number of passive smokers, thus increasing the population at risk of suffering the ill effects caused by this exposure.

Two groups of passive smokers in particular deserve special attention: children, firstly, because they do not choose the air they breathe, and workers who are obliged to live together in closed spaces with their smoking colleagues and clients. A segment of this group, that of workers in transportation companies, was benefited by the prohibition of smoking in Brazilian buses and planes, within and outside of Brazil (Law 9294/96 and Decrete 3157/99).

Nonetheless, for employees of bars and restaurants, the exposure to ETS has only been dealt with by making it obligatory to divide the space into smoking and non-smoking sections. This does not resolve the problem, especially because this division is generally within the same environment and because restaurants are the greatest source of exposure to environmental pollution from tobacco products.^7^ Studies made among waiters and bar staff have indicated the potential risks to their health, as well as the effectiveness of control measures for ETS in these environ- ments.^8,9^ Eisner et al.,^10^ 1998 showed that 74% and 77% of the workers in taverns and bars reported respiratory symptoms and mucosa irritation symptoms, respectively. These disappeared in 59% and 78% of the cases after the prohibition of smoking in these establishments, with significant improvements in respiratory function tests. Other studies have shown that the frequently raised argument that such measures influence the profitability of bars, restaurants and even hotels do not appear to be consistent.^11^ From comparisons of tourist rates and hotel receipts before and after measures restricting smoking in restaurants in some American cities, there appeared not to have been any negative effect on the profits of these establishments, with there even being an increase in tourist business.^6^ Finally, there is evidence that the simple separation into smoking and non-smoking areas does not really appear to resolve the problem: Brauer et al.,^12^ 1998, using cadmium markers to evaluate the concentration of suspended particles in restaurants, concluded that the average concentration of particles in restaurants without restrictions on the use of tobacco was 70% greater than in restaurants with partial restrictions, showing the need for more restrictive measures. These measures, however, appear to have little effect when they are the result of voluntary agreements with establishments in the sector. Evidence for this was brought out in a study done in Australia,^13^ which showed the low adherence of Australian restaurateurs to the agreement, leading the authors to give considerable support to the recommendation for a total prohibition of the use of tobacco in restaurants.

The result of the Brazilian study by Laranjeira et al.,^14^ published in this issue of São Paulo Medical Journal/Revista Paulista de Medicina, corroborates the evidence that has been accumulating in the world's literature on the subject. It shows that the average levels of carbon monoxide in the air exhaled by non-smoking waiters after a day of work in restaurants without restrictions on the use of tobacco are 2.5 times higher than the pre-exposure levels. This makes it clear that studies like this need to be stimulated in Brazil, to evaluate the exposure to and the magnitude of the ill-effects of passive smoking, and the problem represented by occupational exposure to ETS, thereby giving support to public policies for control of tobacco abuse. It also shows that our waiters and restaurant workers, like those all over the world, must be protected by more efficacious measures for restricting the ETS, which will only really be solved by the total restriction of the use of tobacco in closed environments.
